# ﻿Complete mitochondrial genomes of two catfishes (Siluriformes, Bagridae) and their phylogenetic implications

**DOI:** 10.3897/zookeys.1115.85249

**Published:** 2022-07-29

**Authors:** Renyi Zhang, Lei Deng, Xiaomei Lv, Qian Tang

**Affiliations:** 1 School of Life Sciences, Guizhou Normal University, 116 Baoshan Road, Guiyang, Guizhou, 550001, China Guizhou Normal University Guiyang China

**Keywords:** bagrid catfish, mitogenome, phylogenetic analysis, *Tachysurusbrachyrhabdion*, *Tachysurusgracilis*

## Abstract

The mitochondrial genome (mitogenome) has been widely used as a molecular marker to investigate phylogenetic analysis and evolutionary history in fish. However, the study of mitogenomes is still scarce in the family Bagridae. In this study, the mitogenomes of *Tachysurusbrachyrhabdion* and *T.gracilis* were sequenced, annotated, and analyzed. The mitogenomes were found to be 16,532 bp and 16,533 bp, respectively, and each contained 37 typical mitochondrial genes, which are 13 protein-coding genes (PCGs), 22 tRNA genes, two rRNA genes, and a control region. All PCGs begin with the codon ATG, except for the cytochrome c oxidase subunit 1 (*COI*) gene, while seven PCGs end with an incomplete termination codon. All tRNA genes can fold into their typical cloverleaf secondary structures, except for tRNA^Ser(AGY)^, which lacks the dihydrouracil arm. The Ka/Ks ratios for all PCGs are far lower than one. Phylogenetic analyses based on Bayesian inference (BI) and maximum likelihood (ML) showed that the two clades in Bagridae excluded *Ritarita*. The monophyly of *Tachysurus* supports previous research and the traditional classification that *Leiocassis*, *Pseudobagrus*, *Pelteobagrus*, and *Tachysurus* belong to one genus (*Tachysurus*). These findings provide a phylogenetic basis for future phylogenetic and taxonomic studies of Bagridae.

## ﻿Introduction

The family Bagridae, commonly known as bagrid catfish, is widely distributed in Asia and Africa, with about 225 species in 19 genera (Fricke et al. 2022). It is one of the most diverse and complicated groups within Siluriformes. Regan (1911) first promoted bagrid fishes as a single-family and named Bagridae, including two subfamilies: Chrysichthyinae and Bagrinae. Based on osteological features, Jayaram (1968) divided Bagridae into five subfamilies: Ritinae, Chiysichthyinae, Bagrinae, Bagroidinae, and Auchenoglaninae. In 1992, Mo (1992) divided the traditional bagrid catfishes into three monophyletic groups: Caroteidae, Austroglanididae, and Bagridae; the latter Bagridae contained two subfamilies, Ritinae and Bagrinae. In the past two decades, molecular phylogenetic studies showed that Bagridae is monophyletic, with only *Rita* excluded (Sullivan et al. 2006; Vu et al. 2018). However, the phylogenetic relationships between different genera of Bagridae are not well understood, such as the monophyly or the validity of *Leiocassis*, *Pseudobagrus*, *Pelteobagrus*, and *Tachysurus*. Recently, *Leiocassis*, *Pseudobagrus*, *Pelteobagrus*, and *Tachysurus* were classified into *Tachysurus* according to morphological analysis (Cheng et al. 2021; Shao et al. 2021).

*Tachysurusbrachyrhabdion* Cheng, Ishihara & Zhang, 2008 was firstly named *Pseudobagrusbrachyrhabdion* in 2008 (Cheng et al. 2008). It is mainly distributed in the Yuanjiang and Xiangjiang rivers, including the southwest of Hunan Province and the northeast of Guizhou Province in China. *Tachysurusgracilis* Li, Chen & Chan, 2005 was firstly named *Pseudobagrusgracilis* in 2005 (Li et al. 2005). It inhabits in the freshwater drainages of southern China. They are essential economic species of freshwater fishes in local areas.

Mitochondria are eukaryotic organelles that play essential roles in oxidative phosphorylation and other biochemical functions. Similar to other vertebrates, fish mitochondrial DNA (mtDNA) is a circular double-stranded molecule, and it is independent of the nuclear genome (Xiao and Zhang 2000). Fish mtDNA is generally small (15–18 kb), containing 13 protein-coding genes (PCGs), 22 transfer RNA (tRNA) genes, two ribosomal RNA (rRNA) genes, and a non-coding region (D-loop) (Brown 2008; Zhang and Wang 2018; Zhang et al. 2021). The mitochondrial genome has been used to study fish species identification, genome evolution, and phylogenetic studies because of the advantages of its small size, multiple copies, maternal inheritance, rapid evolution rate, and lack of introns (Boore 1999; Xiao and Zhang 2000; Zhang and Wang 2018; Zhang et al. 2021).

In this study, the mitochondrial genomes of two catfishes (*T.brachyrhabdion* and *T.gracilis*) were sequenced, assembled, and compared to reveal their evolutionary relationship. These mitochondrial genomes will provide a phylogenetic basis for future phylogenetic and taxonomic studies of Bagridae.

## ﻿Materials and methods

### ﻿Sample collection and DNA extraction

Specimens of *T.brachyrhabdion* and *T.gracilis* were collected from Jiangkou County (27°46'12"N, 108°46'56"E) and Liping County (26°17'51"N, 109°7'25"E), Guizhou, China, respectively. The samples were preserved in 95% ethanol and stored at -20 °C until DNA extraction. Specimens were recognized as *T.brachyrhabdion* and *T.gracilis* by traditional morphology. The voucher specimens were deposited in the fish specimen room, School of Life Science, Guizhou Normal University under the voucher numbers GZNUSLS201909001~006 and GZNUSLS201907029~030 for *T.brachyrhabdion* and GZNUSLS202005279 for *T.gracilis*. Specimens GZNUSLS201907029 and GZNUSLS202005279 were destroyed for the molecular analysis. Total genomic DNA was extracted from muscle tissues using a standard high salt method (Sambrook et al. 1989). The integrity of the genomic DNA was evaluated via 1% agarose gel electrophoresis, and the concentration and purity of DNA were measured using an Epoch 2 Microplate Spectrophotometer (Bio Tek Instruments, Inc, Vermont, USA).

### ﻿PCR amplification and sequencing

The whole mitogenomes of *Tachysurus* species were amplified in overlapping PCR fragments using 13 primer pairs designed based on the mitogenome of *T.brevicaudatus* (GenBank accession number: NC_021393) by Primer Premier 5.0 software (Lalitha 2000) (Suppl. material 3). PCR amplification were performed as described previously (Zhang et al. 2021). The PCR products were fractionated by electrophoresis through 1% agarose gel electrophoresis. The lengths of fragments were determined by comparison with the DL2000 DNA marker (TaKaRa, Japan). The PCR products were sent to Sangon Biotech. Co., Ltd. (Shanghai, China) for sequencing.

### ﻿Sequence analysis and gene annotation

After sequencing, the sequence fragments were edited and assembled using the SeqMan software of DNAStar (DNASTAR Inc., Madison, WI, USA) to obtain the complete mitogenome sequences. Assembled mitogenome sequences were annotated using the MitoAnnotator on the MitoFish homepage (Sato et al. 2018). tRNA genes and their secondary structures were predicted with MITOS (Bernt et al. 2013) and tRNAscan-SE 2.0 (Chan et al. 2021). The base composition, codon usage, and relative synonymous codon usage (RSCU) values were calculated using MEGA 6.0 (Tamura et al. 2013). Strand asymmetry was calculated using the following formulae: AT-skew = (A-T)/(A+T) and GC-skew = (G-C)/(G+C) (Perna and Kocher 1995). The ratio of nonsynonymous substitutions (Ka), synonymous substitutions (Ks), and evolutionary rates (Ka/Ks) of each PCG was calculated using DnaSP v. 6.0 (Rozas et al. 2017).

### ﻿Phylogenetic analysis

For phylogenetic analysis, sequences of 32 bagrid catfishes were downloaded from GenBank. Additionally, *Cyprinuscarpio* (NC_001606.1), *Silurusasotus* (NC_015806.1), *Liobagrusandersoni* (NC_032035.1), and *L.styani* (NC_034647.1) were used as outgroups. The species used in the analysis are listed in Suppl. material 4. The shared 13 concatenated PCGs were extracted and recombined to construct a matrix using PhyloSuite v.1.1.16 (Zhang et al. 2020). The 13 PCGs were aligned separately using MAFFT v.7.313 (Katoh and Standley 2013) and concatenated into a sequence matrix using PhyloSuite v.1.1.16 (Zhang et al. 2020). The optimal partitioning scheme and nucleotide sequence substitution model of each partition were estimated using PartitionFinder v.2.1.11 (Lanfear et al. 2017) with the Corrected Akaike information criterion (AICc) criteria and greedy algorithm. Bayesian inference (BI) analysis was performed using MrBayes v.3.2.6 (Ronquist et al. 2012) with the models determined by PartitionFinder (Lanfear et al. 2017). Two independent runs of four Markov Chain Monte Carlo (MCMC) chains (one cold chain and three heated chains) were performed for one million generations sampling every 100 generations. The first 25% of the generations was discarded as burnin, and the remaining trees were used to generate a majority rule consensus tree. Maximum likelihood (ML) analysis was carried out using IQ-TREE v.1.6.8 (Nguyen et al. 2015) with 10,000 bootstrap replicates using the ultrafast bootstrapping algorithm (Minh et al. 2013). The phylogenetic trees were visualized and edited using FigTree v1.4.2 (http://tree.bio.ed.ac.uk/software/figtree/).

## ﻿Results

### ﻿Genome structure, organization, and base composition

**Figure 1.**
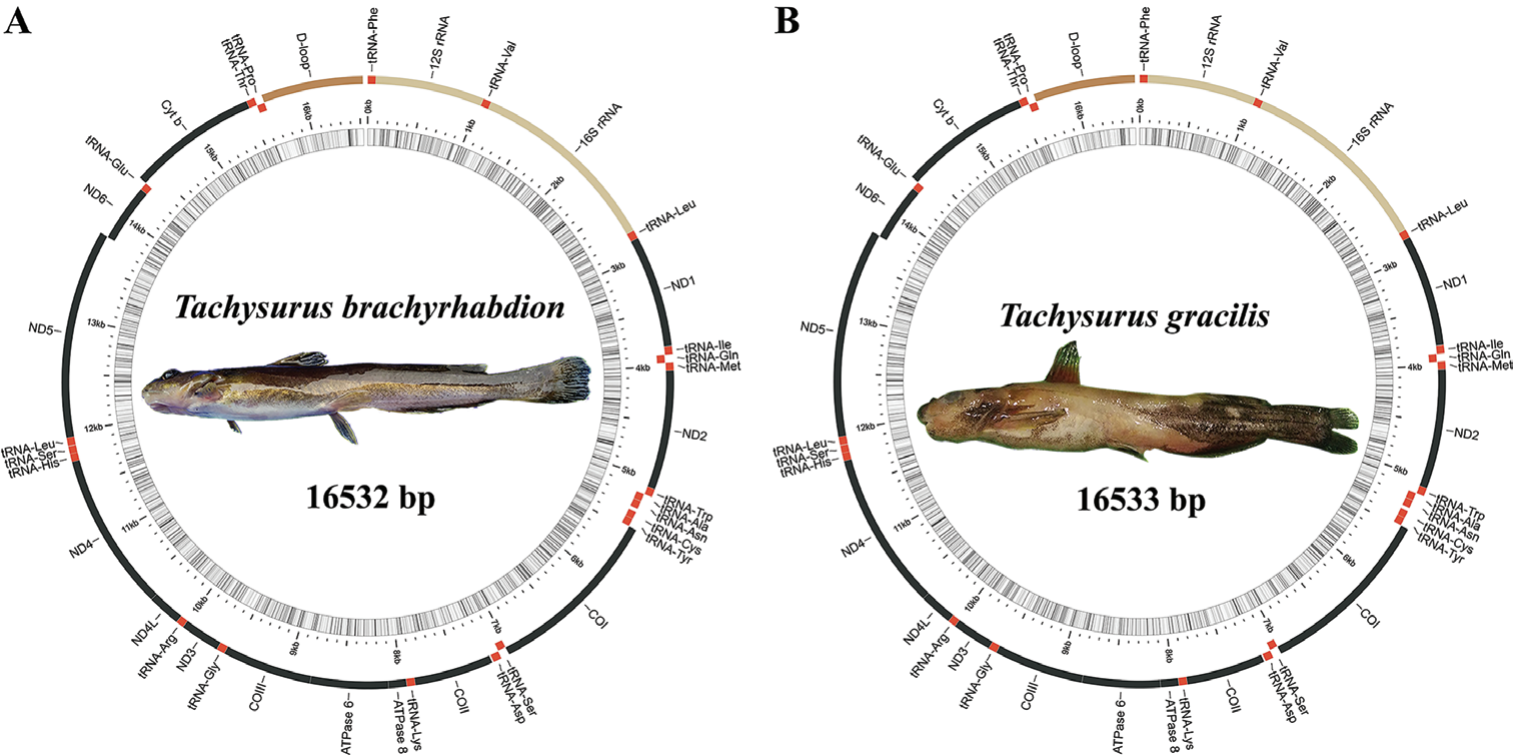
Circular map of the two catfishes mitochondrial genomes.

The entire mitogenome sequences of the two catfishes had lengths of 16,532 bp for *T.brachyrhabdion* and 16,533 bp for *T.gracilis* (GenBank accessions MW712739 and OM759888, respectively) (Fig. 1, Table 1). Both sequences contained 13 PCGs (*ND1-6*, *COI-III*, *ND4L*, *ATP6*, *ATP8*, and *Cytb*), 22 tRNA genes (one for each amino acid, two for Leucine and Serine), two rRNA genes (12S and 16S rRNA), and a non-coding region (D-loop) (Fig. 1, Table 1). Among these genes, one PCG and eight tRNA genes were encoded on the minority strand (L-strand), while another 28 genes were encoded on the majority strand (H-strand) (Fig. 1, Table 1).

**Table 1.** Organization of mitochondrial genome of *T.brachyrhabdion* (TB) and *T.gracilis* (TG). H refers to the majority strand and L refers to the minority strand. Position numbers refer to positions on the majority strand.

Gene	Strand	Nucleotide number		Length (bp)	Intergenic nucleotide		Anticodon	Start/Stop codons	
		TB	TG		TB	TG		Start	Stop
*tRNA ^Phe^*	H	1–70	1–70	70	0	0	GAA		
*12S rRNA*	H	71–1023	71–1023	953	0	0			
*tRNA ^Val^*	H	1024–1095	1024–1095	72	0	0	TAC		
*16S rRNA*	H	1096–2774	1096–2774	1679	0	0			
*tRNA ^Leu (UUR)^*	H	2775–2849	2775–2849	75	0	0	TAA		
*ND1*	H	2850–3824	2850–3824	975	2	2		ATG	TAG
*tRNA ^Ile^*	H	3827–3898	3827–3898	72	-1	-1	GAT		
*tRNA ^Gln^*	L	3898–3968	3898–3968	71	-1	-1	TTG		
*tRNA ^Met^*	H	3968–4037	3968–4037	70	0	0	CAT		
*ND2*	H	4038–5082	4038–5082	1045	0	0		ATG	T
*tRNA ^Trp^*	H	5083–5153	5083–5153	71	2	2	TCA		
*tRNA ^Ala^*	L	5156–5224	5156–5224	69	1	1	TGC		
*tRNA ^Asn^*	L	5226–5298	5226–5298	73	32	32	GTT		
*tRNA ^Cys^*	L	5331–5397	5331–5397	67	0	0	GCA		
*tRNA ^Tyr^*	L	5398–5468	5398–5469	71/72	1	1	GTA		
*COI*	H	5470–7020	5471–7021	1551	0	0		GTG	TAA
*tRNA ^Ser (UCN)^*	L	7021–7091	7022–7092	71	4	4	TGA		
*tRNA ^Asp^*	H	7096–7168	7097–7169	73	14	14	GTC		
*COII*	H	7183–7873	7184–7874	691	0	0		ATG	T
*tRNA ^Lys^*	H	7874–7947	7875–7948	74	1	1	TTT		
*ATPase 8*	H	7949–8116	7950–8117	168	-10	-10		ATG	TAA
*ATPase 6*	H	8107–8789	8108–8790	683	0	0		ATG	TA
*COIII*	H	8790–9573	8791–9574	784	0	0		ATG	T
*tRNA ^Gly^*	H	9574–9647	9575–9648	74	0	0	TCC		
*ND3*	H	9648–9996	9649–9997	349	0	0		ATG	T
*tRNA ^Arg^*	H	9997–10067	9998–10068	71	0	0	TCG		
*ND4L*	H	10068–10364	10069–10365	297	-7	-7		ATG	TAA
*ND4*	H	10358–11738	10359–11739	1381	0	0		ATG	T
*tRNA ^His^*	H	11739–11808	11740–11809	70	0	0	GTG		
*tRNA ^Ser (AGY)^*	H	11809–11875	11810–11876	67	3	3	GCT		
*tRNA ^Leu (CUN)^*	H	11879–11951	11880–11952	73	0	0	TAG		
*ND5*	H	11952–13778	11953–13779	1827	-4	-4		ATG	TAA
*ND6*	L	13775–14290	13776–14291	516	0	0		ATG	TAA
*tRNA ^Glu^*	L	14291–14359	14292–14360	69	2	2	TTC		
*Cytb*	H	14362–15499	14363–15500	1138	0	0		ATG	T
*tRNA ^Thr^*	H	15500–15572	15501–15573	73	-2	-2	TGT		
*tRNA ^Pro^*	L	15571–15640	15572–15641	70	0	0	TGG		
D-loop	H	15641–16532	15642–16533 16533	892	0	0			

The overall base composition for both species was very similar, 31.02% A, 27.05% T, 15.55% G, and 26.38% C for *T.brachyrhabdion* and 31.03% A, 27.14% T, 15.52% G, and 26.31% C for *T.gracilis* (Table 2). The third codon position of PCGs had the highest A+T content (65.75% for *T.brachyrhabdion* and 66.04% for *T.gracilis*), while the first codon position of PCGs had the lowest A+T content (49.00% for *T.brachyrhabdion* and 49.11% for *T.gracilis*) (Table 2). In addition, skew metrics of the mitogenomes showed positive AT-skew and negative GC-skew (Table 2), indicating that As and Cs were more abundant than Ts and Gs.

**Table 2.** Nucleotide composition of the mitochondrial genomes of *T.brachyrhabdion* (TB) and *T.gracilis* (TG).

	Length(bp)		A%		T%		G%		C%		A+T%		AT-skew		GC-skew	
	TB	TG	TB	TG	TB	TG	TB	TG	TB	TG	TB	TG	TB	TG	TB	TG
genome	16532	16533	31.02	31.03	27.05	27.14	15.55	15.52	26.38	26.31	58.07	58.17	0.0683	0.0669	-0.2585	-0.2580
PCGs	11405	11405	28.94	28.99	29.13	29.25	15.37	15.30	26.56	26.46	58.07	58.24	-0.0032	-0.0045	-0.2668	-0.2673
1^st^ codon position	3806	3806	27.06	27.17	21.94	21.94	25.49	25.35	25.51	25.54	49.00	49.11	0.1046	0.1065	-0.0005	-0.0036
2^nd^ codon position	3806	3806	18.61	18.63	40.87	40.95	13.55	13.53	26.97	26.89	59.47	59.58	-0.3743	-0.3746	-0.3312	-0.3307
3^rd^ codon position	3806	3806	41.17	41.17	24.59	24.87	7.05	7.00	27.19	26.95	65.75	66.04	0.2522	0.2467	-0.5880	-0.5876
rRNA	2632	2632	34.77	34.65	22.38	22.34	19.64	19.72	23.21	23.29	57.14	56.99	0.2168	0.2160	-0.0833	-0.0830
tRNA	1566	1567	29.12	29.16	28.10	28.33	22.48	22.34	20.30	20.17	57.22	57.50	0.0179	0.0144	0.0507	0.0511
D-loop region	892	892	31.28	31.17	31.39	31.17	13.79	13.90	23.54	23.77	62.67	62.33	-0.0018	0.0000	-0.2613	-0.2619

### ﻿Protein-coding genes

**Figure 2.**
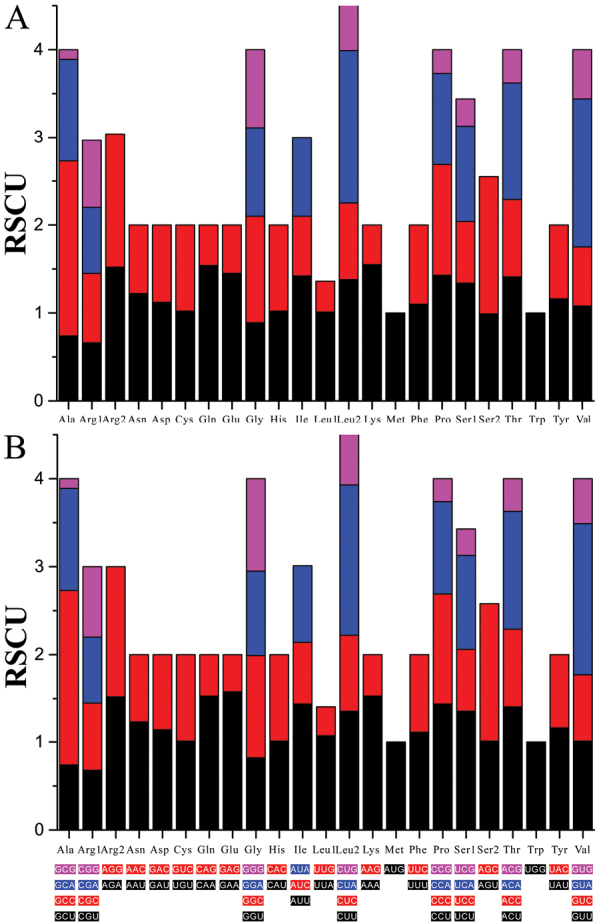
Relative synonymous codon usage (RSCU) in the mitogenomes of the *T.brachyrhabdion***A** and *T.gracilis***B**.

The mitogenomes of *T.brachyrhabdion* and *T.gracilis* had one PCG (*ND6*) encoded on the L-strand and the remaining PCGs on the H-strand. Both mitogenomes had 11,405 bp coding for PCGs, accounting for 3627/3626 amino acids (Table 2, Suppl. material 5). Both species had very similar codon usage, with the most commonly used amino acids being Leu (13.81%, 13.87%), Ser (9.98%, 10.04%), and Ile (8.88%, 8.80%) (Suppl. material 5). The RSCU values of the mitogenomes of the two species are summarized in Fig. 2. All PCGs start with ATG except for *COI*, which used GTG. For these protein-coding genes, the most common stop codon was TAA, although *ND1* used TAG and some used the incomplete stop codon T – or TA-.

**Figure 3.**
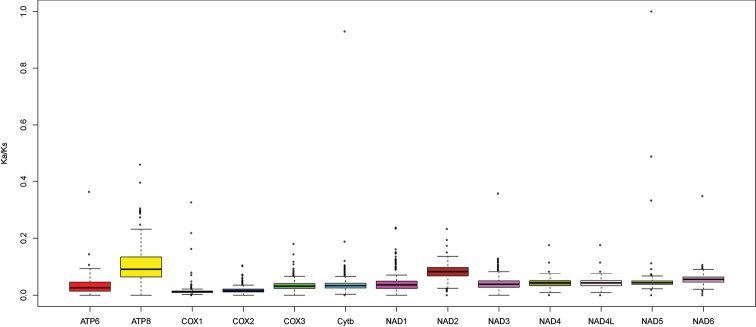
Ka/Ks of each PCG from 33 bagrid catfishes mitogenomes (*Ritarita* was excluded).

To investigate the evolutionary patterns under different selective pressures among 13 PCGs in bagrid catfishes, the value of Ka/Ks was calculated for each PCG, respectively (Fig. 3). The gene (*ATP8*) exhibited the highest ratio of all the PCGs, whereas *COI* had the lowest ratio. However, the Ka/Ks ratios for all PCGs were far lower than one (Fig. 3).

### ﻿Ribosomal, transfer RNA genes and control region

Both the *T.brachyrhabdion* and *T.gracilis* mitogenomes contained two rRNA genes: the large ribosomal RNA subunit (16S rRNA) and small ribosomal RNA subunit (12S rRNA). The 16S rRNA was located between tRNA^Val^ and tRNA^Leu (UUR)^, and the 12S rRNA was located between tRNA^Phe^ and tRNA^Val^. The 12S rRNA genes and the 16S rRNA genes of both mitogenomes were 953 bp and 1679 bp, respectively.

Twenty-one tRNA genes produced the typical cloverleaf secondary structure, while tRNA^Ser (AGY)^ gene lacked the dihydrouracil (DHU) arm (Figs S1 and S2). The sizes of the tRNA genes ranged from 67 bp (tRNA^Cys^ and tRNA^Ser (AGY)^) to 75 bp (tRNA^Leu (UUR)^) in both *T.brachyrhabdion* and *T.gracilis* (Table 1).

The putative control regions were located between *tRNA^Pro^* and *tRNA^Phe^* in the two bagrid catfishes. The control regions of *T.brachyrhabdion* and *T.gracilis* were 892 bp in size. The average A+T content of the CRs (62.33%–62.67%) was higher than that of the whole genomes, PCGs, rRNAs, or tRNAs (57.14%–58.24%) (Table 2).

### ﻿Phylogenetic analysis

**Figure 4.**
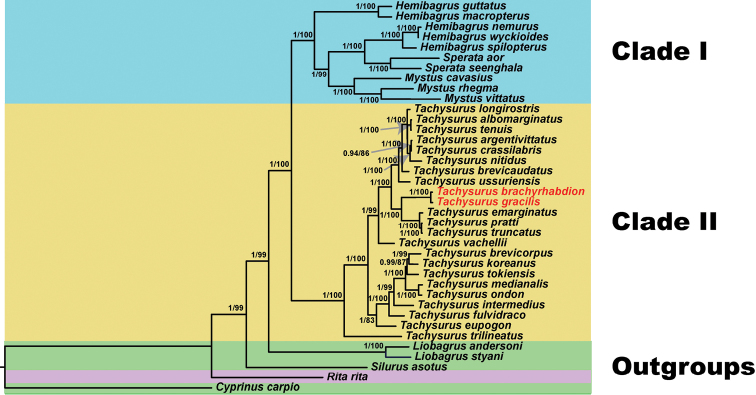
Phylogenetic tree obtained from BI and ML analysis based on the 13 PCGs dataset. The numbers at the nodes separated by “/” indicate the posterior probability (BI) and bootstrap value (ML).

To determine the phylogenetic relationship between *T.brachyrhabdion* and *T.gracilis* in the Characidae, we selected the concatenated nucleotide sequences of the combined mitochondrial gene set (13 PCGs) from 34 bagrid catfishes. As shown in Fig. 4, the phylogenetic analysis of the two tree models (BI and ML) using the combined mitochondrial gene set well supported the tree topologies and yielded identical results. Phylogenetic analysis reveals bagrid catfishes could be separated into two clades excluding *Ritarita* (Hamilton, 1822) with strong support (Fig. 4). Clade I included *Hemibagrus*, *Sperata*, and *Mystus* (BS = 100%, PP = 100%). Clade II was only composed of *Tachysurus* (BS = 100%, PP = 100%). Several monophyletic clades of *Tachysurus*, *Sperata*, *Mystus*, and *Liobagrus* were strongly supported (Fig. 4). Nevertheless, the genus *Hemibagrus* was paraphyletic (Fig. 4). In addition, our phylogenetic trees showed that *T.brachyrhabdion* and *T.gracilis* clustered together forming a group.

## ﻿Discussion

Over the past two to three decades, mitochondrial genes and genomes have been frequently used in fish studies (Xiao and Zhang 2000; Brown 2008; Zhang and Wang 2018). In this study, the complete mitochondrial genomes of *T.brachyrhabdion* and *T.gracilis* were sequenced. The order and arrangement of the two mitogenomes were identical to that of other bagrid catfishes (Liang et al. 2014; Tian et al. 2016; Liu et al. 2019). The genomes had a total length of 16,532 bp and 16,533 bp, with A + T contents at 58.07% and 58.17%, respectively. These A + T biases were within the known range (56.37–59.80%) reported for mitochondrial genomes in closely related fishes (Tian et al. 2016; Liu et al. 2019).

Most of the PCGs of these two species had ATG as the start codon except *COI* that had GTG as the start codon. The *COI* gene usually uses GTG as the start codon in other fishes, such as *Lateolabrax*, *Sinocyclocheilusmultipunctatus*, and *Microphysogobioelongatus* (Shan et al. 2016; Zhang and Wang 2018; Zhang et al. 2021). The PCGs of these two bagrid catfishes had an incomplete stop codon that was automatically filled by post-transcriptional polyadenylation (Ojala et al. 1981). This was a common character in metazoans mitogenomes (Shan et al. 2016; Jiang et al. 2019; Li et al. 2021). Twenty-one tRNA genes showed the typical cloverleaf secondary structure (Suppl. material 1, 2), while the tRNA-Ser^AGY^ gene lacked the dihydrouracil (DHU) arm as noted in other fish species (Zhang and Wang 2018; Zhang et al. 2021). The low Ka/Ks value for each PCG indicated that they were all under strong purifying selection. The DNA barcoding gene *COI* had the lowest evolutionary rate, consistent with the results observed from other fish groups (Sun et al. 2021; Yu et al. 2021).

Phylogenetic analysis revealed Bagridae was supported as a monophyletic group with *Ritarita* excluded (Fig. 4), which is consistent with previous phylogenetic studies (Sullivan et al. 2006; Vu et al. 2018). The phylogenetic tree suggested the classification of *R.rita* should be further revised and perfected. Clade I was composed of two monophyletic groups (*Sperata* and *Mystus*) and one paraphyletic group (*Hemibagrus*) (Fig. 4), which is consistent with the previous study of Liu et al. (2019). The traditional genera *Leiocassis*, *Pseudobagrus*, *Pelteobagrus*, and *Tachysurus* were not each monophyletic (Hardman 2005; Ku et al. 2007; Liu et al. 2019), but instead were grouped into *Tachysurus* as found in recent research (Cheng et al. 2021; Shao et al. 2021). Our results supported that the species in *Leiocassis*, *Pseudobagrus*, *Pelteobagrus*, and *Tachysurus* belong to the same genus, which by taxonomic priority should be *Tachysurus*. Thus, the current concept of *Tachysurus* includes the traditional genera *Leiocassis*, *Pseudobagrus*, *Pelteobagrus*, and *Tachysurus*. Due to limited sample size and molecular data, the phylogeny discussed in this research should be regarded as preliminary. The growing number of available mitogenomes will improve our understanding of the phylogeny and classification of bagrid catfish.

## ﻿Conclusions

This study reported the complete mitochondrial genome sequences of two bagrid catfishes. The study showed that mitogenomes of Bagridae were conserved in structure, gene order, and nucleotide composition. Phylogenetic analysis confirmed that Bagridae is monophyletic group with *Ritarita* excluded and the traditional classification that *Leiocassis*, *Pseudobagrus*, *Pelteobagrus*, and *Tachysurus* belong to one genus.

## Supplementary materials

Supplementary material 1 Figure S1. Predicted tRNA structures of *Tachysurusbrachyrhabdion* Data type image https://binary.pensoft.net/file/721601This dataset is made available under the Open Database License (http://opendatacommons.org/licenses/odbl/1.0/). The Open Database License (ODbL) is a license agreement intended to allow users to freely share, modify, and use this Dataset while maintaining this same freedom for others, provided that the original source and author(s) are credited.Renyi Zhang, Lei Deng1, Xiaomei Lv, Qian Tang

Supplementary material 2 Figure S2. Predicted tRNA structures of *Tachysurusgracilis* Data type image https://binary.pensoft.net/file/721602This dataset is made available under the Open Database License (http://opendatacommons.org/licenses/odbl/1.0/). The Open Database License (ODbL) is a license agreement intended to allow users to freely share, modify, and use this Dataset while maintaining this same freedom for others, provided that the original source and author(s) are credited.Renyi Zhang, Lei Deng1, Xiaomei Lv, Qian Tang

Supplementary material 3 Table S1. Primers used for PCR Data type molecular data https://binary.pensoft.net/file/721603This dataset is made available under the Open Database License (http://opendatacommons.org/licenses/odbl/1.0/). The Open Database License (ODbL) is a license agreement intended to allow users to freely share, modify, and use this Dataset while maintaining this same freedom for others, provided that the original source and author(s) are credited.Renyi Zhang, Lei Deng1, Xiaomei Lv, Qian Tang

Supplementary material 4 Tables S2. Species, GenBank accession number and length of mitogenomes used in this study Data type molecular data https://binary.pensoft.net/file/721604This dataset is made available under the Open Database License (http://opendatacommons.org/licenses/odbl/1.0/). The Open Database License (ODbL) is a license agreement intended to allow users to freely share, modify, and use this Dataset while maintaining this same freedom for others, provided that the original source and author(s) are credited.Renyi Zhang, Lei Deng1, Xiaomei Lv, Qian Tang

Supplementary material 5 Table S3. Number of codons in *T.brachyrhabdion* (TB) and *T.gracilis* (TG) for mitochondrial PCGs Data type molecular data https://binary.pensoft.net/file/721605This dataset is made available under the Open Database License (http://opendatacommons.org/licenses/odbl/1.0/). The Open Database License (ODbL) is a license agreement intended to allow users to freely share, modify, and use this Dataset while maintaining this same freedom for others, provided that the original source and author(s) are credited.Renyi Zhang, Lei Deng1, Xiaomei Lv, Qian Tang
