# Supplementary material for: ﻿Complete mitochondrial genomes of two catfishes (Siluriformes, Bagridae) and their phylogenetic implications
Source: Zookeys. 2022 Jul 29;1115:103–16. doi: 10.3897/zookeys.1115.85249 (PMC9848681; doi:10.3897/zookeys.1115.85249)
Supplement: Supplementary material 3 — Table S1. Primers used for PCR [file zookeys-1115-103_article-85249__-s003.docx]

**Table S1** Primers used for PCR.

| **Primer name** | **Primer sequence** | **Annealing T (**°C**)** |
| --- | --- | --- |
| PF1 | 5′- ACCTCACCACTTCTTGTTTTC-3′ | 47°C |
| PR1 | 5′- TTGTAGTTTTATGTCCGCTTCT-3′ |  |
| PF2 | 5′- AAGGAACTCGGCAAACAC-3′ | 47°C |
| PR2 | 5′- CGGCGTATTCTACATTGA-3′ |  |
| PF3 | 5′- CAACACAAGAAGCAGTATGA-3′ | 44°C |
| PR3 | 5′- TTTGGTTTGTGTGTTTTGTA-3′ |  |
| PF4 | 5′-TTATCGGGGGATGAGGGG-3′ | 47°C |
| PR4 | 5′-TGTTGATAGAGGATGGGGTCCC-3′ |  |
| PF5 | 5′-AAGCCCCCTGCCATCTCTCA-3′ | 54°C |
| PR5 | 5′-CCGGGGACTAGGTCTTGTGTTGG-3′ |  |
| PF6 | 5′-TAATGGCACATCCCTCAC-3′ | 48°C |
| PR6 | 5′-TTACGGCTACTGCTACTTCT-3′ |  |
| PF7 | 5′-GCCACCTCCTACCAGAAG-3′ | 49°C |
| PR7 | 5′-GTCATAAGGGAATCACGGA-3′ |  |
| PF8 | 5′-CCCTACGAATGCGGTTTCGA-3′ | 51°C |
| PR8 | 5′-GTTCGGCTGTGGGTTCGTTC-3′ |  |
| PF9 | 5′-CCGTAGCAGGCTCAATAGT-3′ | 50°C |
| PR9 | 5′-TAGAGCTGATACTGGGGTTG-3′ |  |
| PF10 | 5′-AGCAATAATCACCTTAGTCAC-3′ | 43°C |
| PR10 | 5′-GTTTTGTTATTATGTCTTTTTGT-3′ |  |
| PF11 | 5′-AGAAATTGGACCAAAAGGT-3′ | 49°C |
| PR11 | 5′-GTGAGAAAAGTGCTAGGGAT-3′ |  |
| PF12 | 5′-ACAACGCAACACTAACACG-3′ | 48°C |
| PR12 | 5′-CAAGCAAGGAAATGGACTC-3′ |  |
| PF13 | 5′-AGTAAGAGACCACCAACCCT-3′ | 50°C |
| PR13 | 5′-GCTAAATCCACCTTCAGACAT-3′ |  |
