# Supplementary material for: ﻿Complete mitochondrial genomes of two catfishes (Siluriformes, Bagridae) and their phylogenetic implications
Source: Zookeys. 2022 Jul 29;1115:103–16. doi: 10.3897/zookeys.1115.85249 (PMC9848681; doi:10.3897/zookeys.1115.85249)
Supplement: Supplementary material 4 — Tables S2. Species, GenBank accession number and length of mitogenomes used in this study [file zookeys-1115-103_article-85249__-s004.docx]

**Table S3** The number of codons in *T. brachyrhabdion* (TB) and *T*. *gracilis* (TG) mitochondrial PCGs.

| Codon | TB | TG | Codon | TB | TG | Codon | TB | TG | Codon | TB | TG |
| --- | --- | --- | --- | --- | --- | --- | --- | --- | --- | --- | --- |
| UUU(F) | 84 | 86 | UCU(S) | 81 | 82 | UAU(Y) | 110 | 111 | UGU(C) | 63 | 62 |
| UUC(F) | 69 | 69 | UCC(S) | 42 | 43 | UAC(Y) | 80 | 80 | UGC(C) | 60 | 61 |
| UUA(L) | 84 | 90 | UCA(S) | 66 | 65 | UAA(*) | 79 | 81 | UGA(*) | 67 | 66 |
| UUG(L) | 29 | 28 | UCG(S) | 19 | 18 | UAG(*) | 28 | 28 | UGG(W) | 50 | 50 |
| CUU(L) | 115 | 113 | CCU(P) | 105 | 104 | CAU(H) | 77 | 76 | CGU(R) | 29 | 30 |
| CUC(L) | 73 | 73 | CCC(P) | 92 | 90 | CAC(H) | 74 | 75 | CGC(R) | 35 | 34 |
| CUA(L) | 145 | 143 | CCA(P) | 76 | 76 | CAA(Q) | 74 | 72 | CGA(R) | 33 | 33 |
| CUG(L) | 55 | 56 | CCG(P) | 20 | 19 | CAG(Q) | 22 | 22 | CGG(R) | 34 | 35 |
| AUU(I) | 152 | 153 | ACU(T) | 97 | 99 | AAU(N) | 137 | 138 | AGU(S) | 60 | 61 |
| AUC(I) | 73 | 74 | ACC(T) | 61 | 63 | AAC(N) | 87 | 86 | AGC(S) | 94 | 95 |
| AUA(I) | 97 | 92 | ACA(T) | 92 | 95 | AAA(K) | 75 | 74 | AGA(R) | 67 | 67 |
| AUG(M) | 40 | 42 | ACG(T) | 26 | 26 | AAG(K) | 22 | 23 | AGG(R) | 67 | 65 |
| GUU(V) | 21 | 20 | GCU(A) | 35 | 34 | GAU(D) | 19 | 20 | GGU(G) | 31 | 28 |
| GUC(V) | 13 | 15 | GCC(A) | 94 | 91 | GAC(D) | 15 | 15 | GGC(G) | 42 | 40 |
| GUA(V) | 33 | 34 | GCA(A) | 55 | 53 | GAA(E) | 32 | 34 | GGA(G) | 35 | 33 |
| GUG(V) | 11 | 10 | GCG(A) | 5 | 5 | GAG(E) | 12 | 9 | GGG(G) | 31 | 36 |
