# Supplementary material for: ﻿Complete mitochondrial genomes of two catfishes (Siluriformes, Bagridae) and their phylogenetic implications
Source: Zookeys. 2022 Jul 29;1115:103–16. doi: 10.3897/zookeys.1115.85249 (PMC9848681; doi:10.3897/zookeys.1115.85249)
Supplement: Supplementary material 5 — Table S3. Number of codons in T.brachyrhabdion (TB) and T.gracilis (TG) for mitochondrial PCGs [file zookeys-1115-103_article-85249__-s005.docx]

**Tables S2** Species, GenBank accession number and length of mitogenomes used in this study.

| Organism | Family | Genus | Length (bp) | Accession No. |
| --- | --- | --- | --- | --- |
| *Cyprinus carpio* | Cyprinidae | *Cyprinus* | 16575 | NC_001606.1 |
| *Hemibagrus guttatus* | Bagridae | *Hemibagrus* | 16528 | NC_023976.1 |
| *Hemibagrus macropterus* | Bagridae | *Hemibagrus* | 16530 | NC_019592.1 |
| *Hemibagrus nemurus* | Bagridae | *Hemibagrus* | 16526 | KM454860.1 |
| *Hemibagrus spilopterus* | Bagridae | *Hemibagrus* | 16521 | NC_023222.1 |
| *Hemibagrus wyckioides* | Bagridae | *Hemibagrus* | 16525 | NC_024278.1 |
| *Liobagrus andersoni* | Amblycipitidae | *Liobagrus* | 16514 | NC_032035.1 |
| *Liobagrus styani* | Amblycipitidae | *Liobagrus* | 16515 | NC_034647.1 |
| *Mystus cavasius* | Bagridae | *Mystus* | 16554 | NC_030187.1 |
| *Mystus rhegma* | Bagridae | *Mystus* | 16525 | NC_023223.1 |
| *Mystus vittatus* | Bagridae | *Mystus* | 16244 | NC_032082.1 |
| *Rita rita* | Bagridae | *Rita* | 16449 | NC_023376.1 |
| *Silurus asotus* | Siluridae | *Silurus* | 16521 | NC_015806.1 |
| *Sperata aor* | Bagridae | *Sperata* | 16555 | KX950699.1 |
| *Sperata seenghala* | Bagridae | *Sperata* | 16588 | AB907556.1 |
| *Tachysurus albomarginatus* | Bagridae | *Tachysurus* | 16533 | NC_022726.1 |
| *Tachysurus argentivittatus* | Bagridae | *Tachysurus* | 16534 | NC_030538.1 |
| *Tachysurus brachyrhabdion* | Bagridae | *Tachysurus* | 16532 | MW712739 |
| *Tachysurus brevicaudatus* | Bagridae | *Tachysurus* | 16533 | NC_021393.1 |
| *Tachysurus brevicorpus* | Bagridae | *Tachysurus* | 16526 | NC_015625.1 |
| *Tachysurus crassilabris* | Bagridae | *Tachysurus* | 16534 | NC_021394.1 |
| *Tachysurus dumeril* | Bagridae | *Leiocassis* | 16534 | NC_014586.1 |
| *Tachysurus emarginatus* | Bagridae | *Tachysurus* | 16534 | NC_024279.1 |
| *Tachysurus eupogon* | Bagridae | *Tachysurus* | 16562 | NC_018768.1 |
| *Tachysurus fulvidraco* | Bagridae | *Tachysurus* | 16527 | NC_015888.1 |
| *Tachysurus gracilis* | Bagridae | *Tachysurus* | 16533 | OM759888 |
| *Tachysurus intermedius* | Bagridae | *Tachysurus* | 16532 | KY962416.1 |
| *Tachysurus koreanus* | Bagridae | *Tachysurus* | 16532 | NC_028434.1 |
| *Tachysurus medianalis* | Bagridae | *Tachysurus* | 16647 | NC_037048.1 |
| *Tachysurus nitidus* | Bagridae | *Tachysurus* | 16532 | NC_014859.1 |
| *Tachysurus ondon* | Bagridae | *Tachysurus* | 16534 | NC_022725.1 |
| *Tachysurus pratti* | Bagridae | *Tachysurus* | 16533 | NC_041443.1 |
| *Tachysurus tenuis* | Bagridae | *Tachysurus* | 16535 | NC_035498.1 |
| *Tachysurus tokiensis* | Bagridae | *Tachysurus* | 16529 | NC_004697.1 |
| *Tachysurus trilineatus* | Bagridae | *Tachysurus* | 16535 | NC_022705.1 |
| *Tachysurus truncatus* | Bagridae | *Tachysurus* | 16533 | NC_021395.1 |
| *Tachysurus ussuriensis* | Bagridae | *Tachysurus* | 16536 | NC_020344.1 |
| *Tachysurus vachellii* | Bagridae | *Tachysurus* | 16527 | NC_014862.1 |
